# P53 activation inhibits all types of hematopoietic progenitors and all stages of megakaryopoiesis

**DOI:** 10.18632/oncotarget.7881

**Published:** 2016-03-03

**Authors:** Emna Mahfoudhi, Larissa Lordier, Caroline Marty, Jiajia Pan, Anita Roy, Lydia Roy, Philippe Rameau, Salem Abbes, Najet Debili, Hana Raslova, Yunhua Chang, Laurent Debussche, William Vainchenker, Isabelle Plo

**Affiliations:** ^1^ INSERM, UMR 1170, Laboratory of Excellence GR-Ex, Villejuif, France; ^2^ UMR 1170, Université Paris-Saclay, Gustave Roussy, Villejuif, France; ^3^ Gustave Roussy, Villejuif, France; ^4^ Laboratory of Excellence GR-Ex, Villejuif, France; ^5^ Laboratoire d'Hématologie Moléculaire et Cellulaire, Institut Pasteur de Tunis, Université de Tunis El Manar, Belvédère, Tunisie; ^6^ Départment of Clinical Hematology, Hôpital Henri-Mondor, Créteil, France; ^7^ Flow Cytometry Platform, Gustave Roussy, Villejuif, France; ^8^ Sanofi Oncology, Vitry-sur-Seine, France

**Keywords:** p53, progenitors, MDM2 inhibitors, megakaryopoiesis, apoptosis

## Abstract

*TP53* also known as p53 is a tumor suppressor gene mutated in a variety of cancers. P53 is involved in cell cycle, apoptosis and DNA repair mechanisms and is thus tightly controlled by many regulators. Recently, strategies to treat cancer have focused on the development of MDM2 antagonists to induce p53 stabilization and restore cell death in p53 non-mutated cancers. However, some of these molecules display adverse effects in patients including induction of thrombocytopenia. In the present study, we have explored the effect of SAR405838 not only on human megakaryopoiesis but also more generally on hematopoiesis. We compared its effect to MI-219 and Nutlin, which are less potent MDM2 antagonists than SAR405838. We found that all these compounds induce a deleterious effect on all types of hematopoietic progenitors, as well as on erythroid and megakaryocytic differentiation. Moreover, they inhibit both early and late stages of megakaryopoiesis including ploidization and proplatelet formation. In conclusion, MDM2 antagonists induced a major hematopoietic defect *in vitro* as well as an inhibition of all stages of megakaryopoiesis that may account for *in vivo* thrombocytopenia observed in treated patients.

## INTRODUCTION

*TP53* is a tumor suppressor gene mutated in numerous cancers, including more than 50% of solid tumors and less than 5% of hematological malignancies. *P53* mutations are loss-of-function affecting the DNA binding or the transactivation domain. P53 plays a key role in cell death regulation, cell cycle checkpoint and DNA repair control by regulating the expression of many genes such as *P21*, *GADD45* and pro-apoptotic genes including *PUMA*, *NOXA* and the Bcl-2 family member called *BAX* [1, 2]. P53 protein levels are maintained at low levels due to a tight regulation by the ubiquitin ligase MDM2. In response to a cellular stress, the level and activity of p53 rise due to its stabilization following MDM2 degradation. Recently, strategies of treatment based on the stabilization of p53 have been considered in p53 non-mutated cancers and aim at disrupting the interaction between MDM2 and p53 [3]. Thus, many MDM2 antagonists with various activities have been designed for solid tumors as well as hematological cancers [4–7]. Clinical and preclinical data in rats and monkeys have shown that some MDM2 antagonists such as Nutlin or the RG7112 compound led to a major thrombocytopenia associated with a neutropenia or, less frequently, with a mild anemia in monkeys [6, 8]. Similar adverse effects were found with the SAR405838 compound, which showed a hematopoietic toxicity with a reversible thrombocytopenia [9]. *In vitro* studies have also demonstrated that these molecules impair megakaryopoiesis mainly at the progenitor level, but also directly decrease mature megakaryocytes (MKs) and platelet production [8]. All these observations suggest that MDM2 antagonists profoundly affect megakaryopoiesis.

Megakaryopoiesis is a unique model of differentiation that combines two specific features: a physiological polyploidization of the bone marrow precursors known as MKs, and their cytoplasmic fragmentation at the end of their differentiation to produce mature circulating blood platelets [10, 11]. Ploidization is related to a process called endomitosis, which is a consequence of defective cytokinesis and karyokinesis [12–14]. The modal MK ploidy in the marrow is 16N and the ploidy level can reach 64N or more, indicating that the MK cell cycle is not blocked at 4N [15, 16]. By increasing the size of the genome and the size of the cell at each round of DNA duplication [17, 18], polyploidization increases platelet production [16]. BAX has been found to play a major role in the p53-induced death by apoptosis of the MKs [19], proplatelet (PPT) formation and platelet shedding [19, 20].

In the present study, we have explored the effect of SAR405838 not only on human megakaryopoiesis but also more generally on hematopoiesis. Moreover, we have compared its effect to the MI-219 compound, which is a less potent MDM2 antagonist than SAR405838 [21]. Specifically, we have studied their effect on hematopoietic progenitors, MK differentiation, ploidization and PPT formation.

## RESULTS

### Effect of p53 stabilization on CD34^+^ progenitors

**Figure 1:**
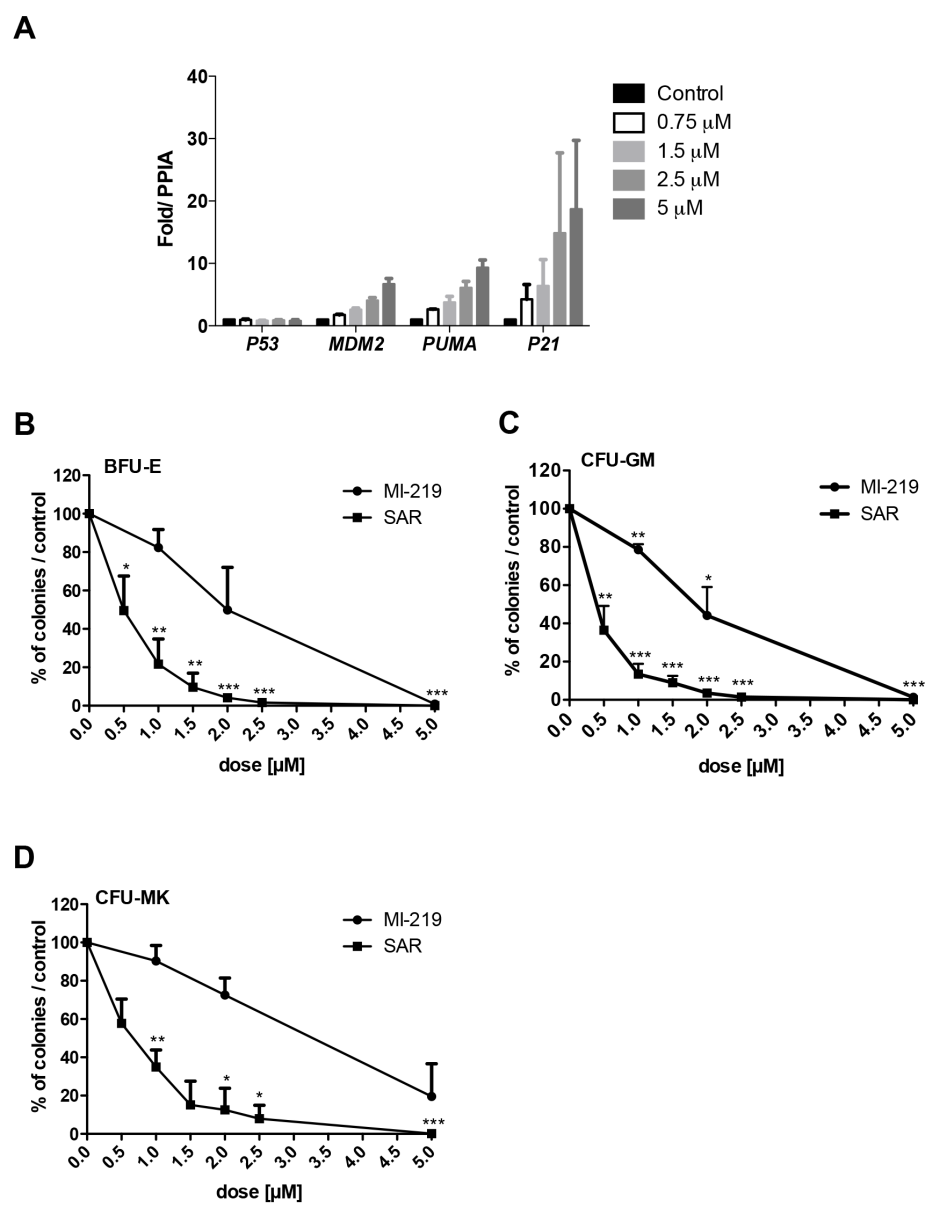
Effects of SAR405838 on CD34+ progenitors **A.** Dose-effect of SAR405838 on p53 target genes by qRT-PCR. CD34^+^ cells were treated or not with SAR405838 at various doses (0.75 M, 1.5 μM, 2.5 μM and 5 μM). The expression of *P53* and its targets *(MDM2*, *PUMA*, *P21*) were quantified by qRT-PCR related to *PPIA*. Results are the mean ± SEM of 2 independent experiments in duplicate. **B, C, D.** Cells were seeded in methylcellulose (B, C) or in fibrin clot (D) in the presence of MI-219 or SAR405838 to assay the number of BFU-E, CFU-GM and CFU-MK progenitors. Results are presented as percentage compared to control. Results represent the mean of 3 experiments for BFU-E and CFU-GM and 3 experiments for CFU-MK made in triplicate. * represents statistical significance using student *t* test (* P<0.05; **P<0.01; ***P<0.001).

To investigate the impact of p53 stabilization on hematopoietic progenitors, CD34^+^ cells were cultured for one day in serum-free medium with TPO and SCF in the presence of the MDM2 antagonist SAR405838 before quantifying the p53 target genes by qRT-PCR. As shown in Figure 1A, SAR405838 induced a dose-dependent effect on *MDM2*, *PUMA* and *P21* expression confirming a functional p53 in these cells.

The effect of increasing concentrations of SAR405838 on the number of progenitors was tested by colony assays and compared to MI-219, a less potent MDM2 antagonist. SAR405838 induced a significant dose-dependent reduction of all the progenitors including the BFU-E (Burst Forming Unit-Erythroid), the CFU-GM (Colony Forming Unit-Granulocyte Macrophage) and the CFU-MK (Colony Forming Unit-Megakaryocyte) compared to untreated control cells. SAR405838 induced a 50% reduction of BFU-E, CFU-GM and CFU-MK numbers at a 0.5-1 μM dose while MI-219 has a significant less potent activity compared to SAR405838 (Figure 1B/1C/1D).

**Figure 2:**
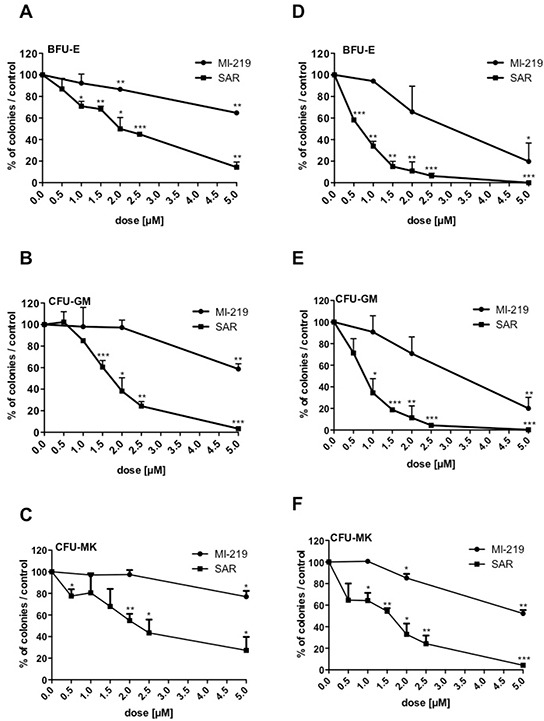
Irreversibility of SAR405838 on CD34+ progenitors **A, B, C, D, E, F.** Cells were treated with various doses of MI-219 or SAR405838 for 3 days (A, B, C) or 5 days (D, E, F), washed and seeded in methylcellulose (A, B, D, E) or in fibrin clot (C, F) to assay the number of BFU-E, CFU-GM and CFU-MK progenitors. Results are presented as percentages compared to control. The results represent the mean of 2 experiments made in triplicate. * represents statistical significance (* P<0.05; **P<0.01; ***P<0.001).

To study the time-response effect of SAR405838 and its reversibility, CD34^+^ cells were seeded in serum-free medium supplemented with cytokines and exposed for 3 or 5 days to either SAR405838 or MI-219 at different concentrations. The treated cells were washed and plated in colony assay to assess the number of progenitors. A 3-day SAR405838 treatment induced a reduction of all progenitors (BFU-E, CFU-GM and CFU-MK) decreasing even further after 5 days of treatment. IC50 were around 2 μM after 3 days of treatment (Figure 2A/2B/2C) and 0.5 to 1 μM after 5 days (Figure 2D/2E/2F). These results strongly suggest that the deleterious effect of MDM2 antagonist is related to the number of cell division and is not reversible.

**Figure 3:**
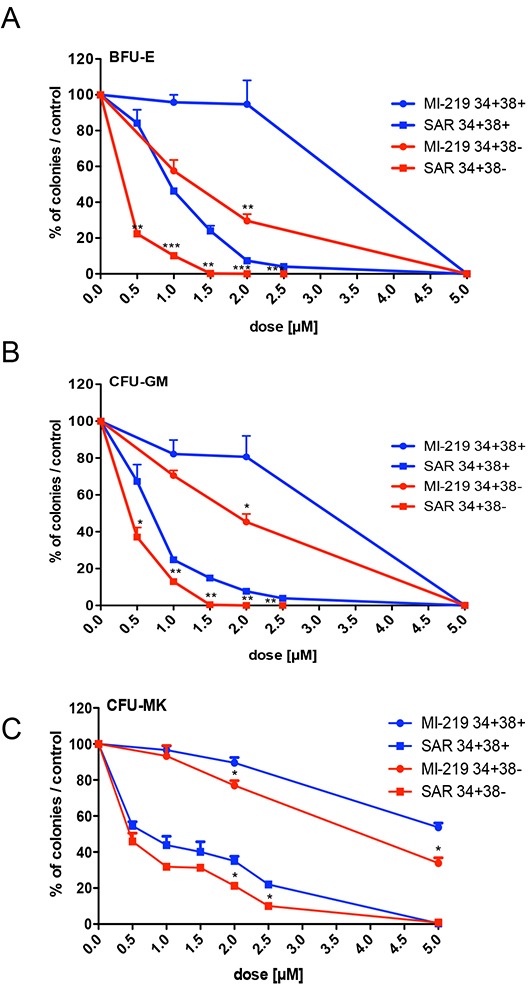
Effect of SAR405838 on immature and committed progenitors **A, B, C.** Cells were sorted either as CD34^+^CD38^−^ immature cells or as CD34^+^CD38^+^ committed cells. They were seeded in methylcellulose (A, B) or in fibrin clot (C) in the presence of various doses of MI-219 or SAR405838 to assay the number of BFU-E, CFU-GM and CFU-MK progenitors. Results are presented as percentages compared to control. The results represent the mean of 2 experiments made in triplicate * represents statistical significance between CD34^+^CD38^+^ and CD34^+^CD38^−^ cells (* P<0.05; **P<0.01; ***P<0.001).

Then, we tested the dose-effect of SAR405838 on immature CD34^+^CD38^−^ progenitors (a population enriched in hematopoietic stem cells) in comparison to the more mature CD34^+^CD38^+^ progenitors. The CD34^+^CD38^−^ progenitors were more sensitive to SAR405838 than the CD34^+^CD38^+^ progenitors, especially BFU-E by 2-fold (Figure 3A/3B/3C).

Altogether, p53 activation induced an irreversible deleterious effect of all hematopoietic progenitors including immature progenitors.

### Effects of p53 activation during erythroid differentiation

**Figure 4:**
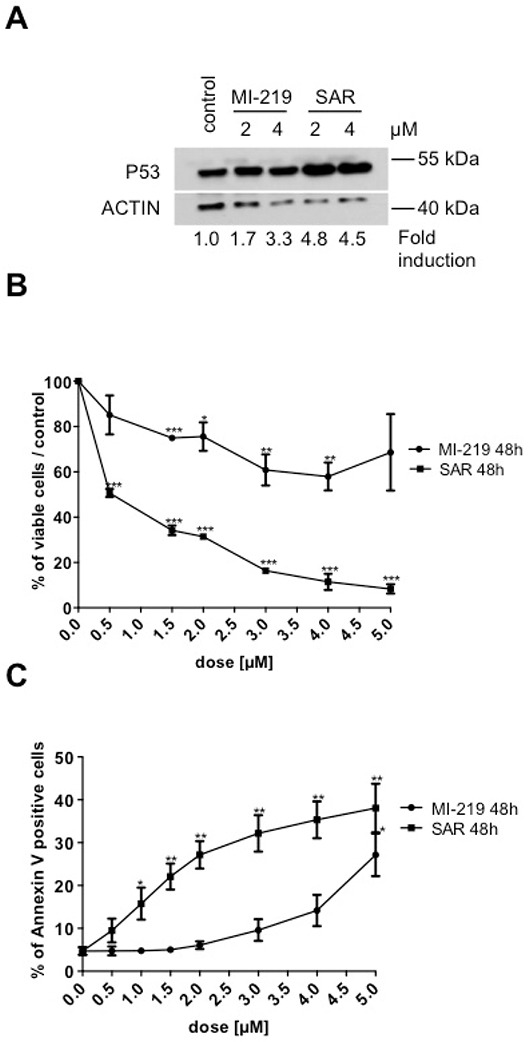
Effect of SAR405838 on erythroid differentiation **A.** Day 8-erythroblasts treated with MI-219 or SAR405838 were analyzed for p53 expression by western-blot. β-actin was used as loading control. **B.** Day 8-erythroblasts were treated with various doses of MI-219 or SAR405838 for 48 hours and counted by trypan blue exclusion. **C.** Day 8-erythroblasts were treated with various doses of MI-219 or SAR405838 for 48 hours and apoptotic cells were analyzed by flow cytometry using Annexin V assay. The results represent the mean of 3 independent experiments. * represents statistical significance (*P<0.05; **P<0.01; ***P<0.001).

The effect of p53 stabilization was explored during erythroid differentiation. For that purpose, CD34^+^ progenitors were cultured in serum-free medium with SCF and EPO for 8 days. SAR405838- or MI-219-treated erythroblasts were analyzed for p53 expression by western-blot. As shown in Figure 4A, in comparison to the less potent MI-219, SAR405838 induced a more important increase in p53 expression compared to untreated cells. Day 8-erythroblasts were treated with various doses of SAR405838 or MI-219 for 48 hours. The viable cells were counted by Trypan blue exclusion and the apoptotic cells were quantified by flow cytometry analysis using annexin V/ propidium iodide labeling. As shown in the Figure 4B/4C, SAR405838 induced a great decrease in viable cells compared to MI-219 with a 0.5 μM IC50. Concomitantly, apoptotic cells increased in a dose-dependent manner with a 48 hour SAR405838 treatment as low as 0.5 μM.

Altogether these results showed that SAR405838 by p53 stabilization has a cytotoxic effect on the erythroid lineage, which is as sensitive as progenitors.

### Effects of p53 stabilization on the viability of CD34^+^ progenitors at early stages of megakaryocytic differentiation

**Figure 5:**
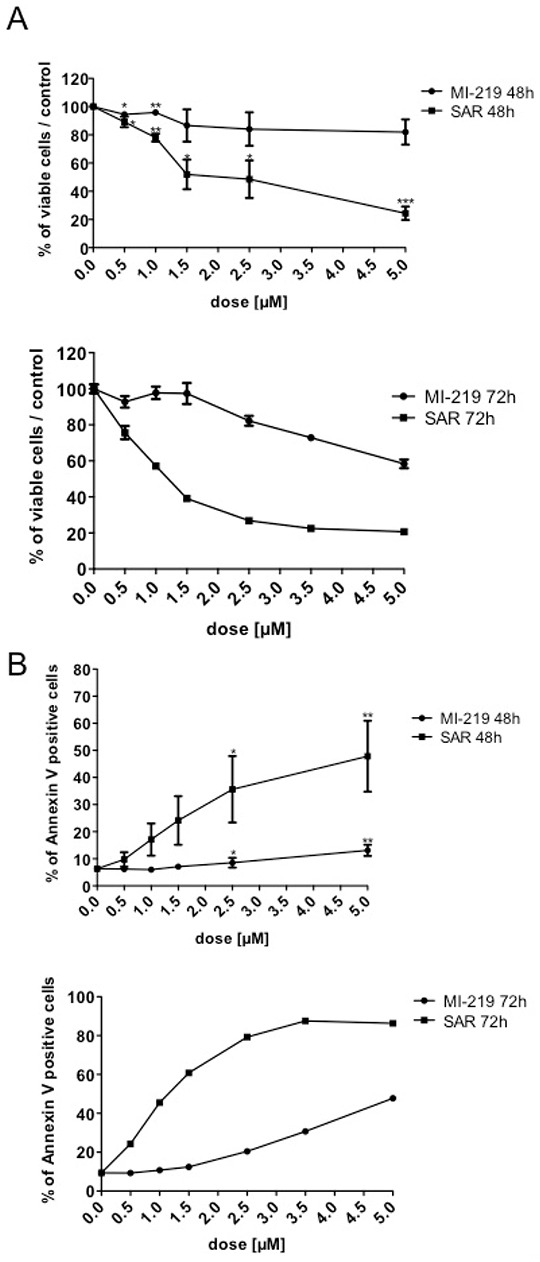
Effect of SAR405838 on early stage of megakaryocytic differentiation CD34^+^ cells were cultured for one day and then treated with various doses of MI-219 or SAR405838 for 48 hours or 72 hours. **A.** Cells were counted by Trypan blue exclusion. **B.** Apoptotic cells were analyzed by flow cytometry using Annexin V assay. The results represent the mean of 3 independent experiments. * represents statistical significance (*P<0.05; **P<0.01; ***P<0.001)

We explored the effect of p53 stabilization on the viability of CD34^+^progenitors at early stages of megakaryocytic differentiation. CD34^+^ progenitors were cultured for one day in serum-free medium supplemented with SCF and TPO and treated with SAR405838 or MI-219. After a 48 or 72 hour incubation, cells were counted by Trypan blue exclusion and apoptotic cells were quantified by flow cytometry using Annexin V / Propidium iodide labeling. SAR405838 induced a significant decrease in the number of viable cells compared to MI-219 or control and the IC50 was found to be around 1.5 μM at 48 hours and 72 hours of SAR405838 treatment (Figure 5A). Concomitantly, apoptotic cells increased in a dose-dependent manner for 48 hours of 1 μM SAR405838 treatment.

Altogether these results confirmed previous findings showing that p53 stabilization has a cytotoxic effect on progenitor cells harboring early stages of MK differentiation at a similar range of concentrations than found for the inhibition of their cloning efficiency.

### Effects of p53 stabilization during megakaryocytic differentiation

**Figure 6:**
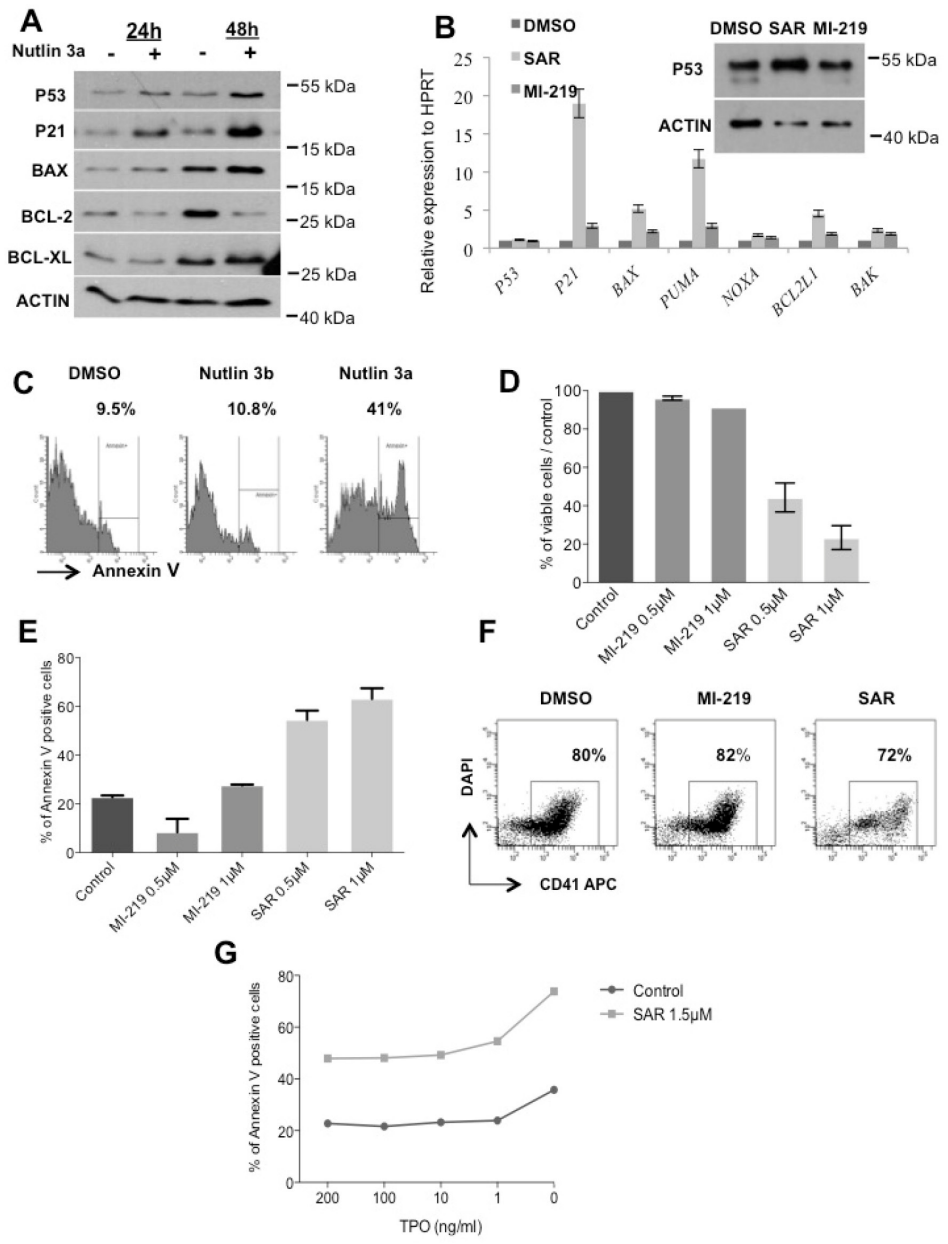
Effect of p53 reactivation on late stage of megakaryocytic differentiation **A.** Day 10-MK treated with Nutlin 3a (5 μM for 24 hours or 48 hours) were analyzed for p53, p21, Bax, Bcl-2, Bcl-xL expression by western-blot. β-actin was used as loading control. **B.** Day 11-MKs treated with MI-219 or SAR405838 (5 μM for 24 hours) were analyzed for p53 expression by western-blot. β-actin was used as loading control. In parallel, the expression of *P53, BAK, BCL2L1, NOXA, PUMA, BAX, P21 and P53* were quantified by qRT-PCR related to *HPRT*. **C.** Day 7-megakaryoblasts were exposed to 5 μM Nutlin 3a or Nutlin 3b for 24 hours and apoptotic cells were analyzed by flow cytometry using Annexin V assay. (n=3). **D, E, F.** Day 6-megakaryoblasts were exposed to MI-219 or SAR405838 (0.5 or 1 μM for 72 hours) and cell viability (D), apoptosis (E) or CD41 expression (F) were investigated. The results represent the mean of 2 experiments. * represents statistical significance (*P<0.05; **P<0.01; ***P<0.001). **G.** Day 6-megakaryoblasts were exposed to SAR405838 (1.5 μM for 48 hours) in the presence of various doses of TPO and apoptotic cells were analyzed by flow cytometry using annexin V assay. One representative experiment (n= 2).

We also explored the effects of p53 stabilization at a later stage of megakaryocytic differentiation. CD34^+^ progenitors were cultured in serum-free medium with SCF and TPO. Day 7-MKs were treated with 5 μM Nutlin 3a for 24 or 48 hours, which led to an increase in p53, p21, BAX, BCL2 and BCL-XL levels, confirming a functional p53 activity (Figure 6A). Similar results were obtained with SAR405838 (1 μM, 24 hours) in day 11-MKs with a significant increase in *P21*, *BAX*, *PUMA* and *BCL2L1* transcripts compared to 1 μM MI-219 or untreated control (Figure 6B). Consequently, we observed an increase in apoptotic cells in day 8-MKs exposed after 24 hours treatment with 5 μM Nutlin 3a but not with Nutlin 3b (an inactive compound) or control (Figure 6C). Similar results were found with day 6-megakaryoblasts treated with two doses of SAR405838 for 72 hours (Figure 6D/6E) without any major effect on the percentage of CD41^+^ cells (Figure 6F). These latter results may suggest that SAR405838 induces cytotoxicity with the same efficiency in all immature cell types during *in vitro* differentiation experiments. As shown in Figure 6D/6E, at the dose of 0.5 μM the SAR405838 induced a 50% decreased in the number of viable cells while the apoptotic cells reached 50% of the total cells. We checked if the effect of SAR405838 on MKs could be reverted by the addition of high TPO doses. CD34^+^ progenitors were cultured in serum-free medium supplemented with SCF and low dose of TPO (1 ng/mL) for 6 days. Day 6-megakaryoblasts were then treated with 1.5 μM of SAR405838 for 48 hours and various doses of TPO. As shown in the Figure 6G, increasing doses of TPO were unable to revert the effect of SAR405838-mediated MK apoptosis demonstrating an irreversible effect of SAR405838.

Altogether these results showed that p53 reactivation has a stronger cytotoxic effect during late stages of megakaryocytic differentiation with a lower IC50 (0.5 μM) than on MK progenitors (2.5 μM).

### Effects of p53 stabilization on late stages of megakaryocyte differentiation

First, we investigated if MDM2 inhibitors affect MK ploidization. SAR405838 induced a significant reduction in the mean ploidy (3.0 N at the dose of 1 μM versus 4.4 N for untreated control or for MI-219 treatment) (Supplementary Figure S1).

**Figure 7:**
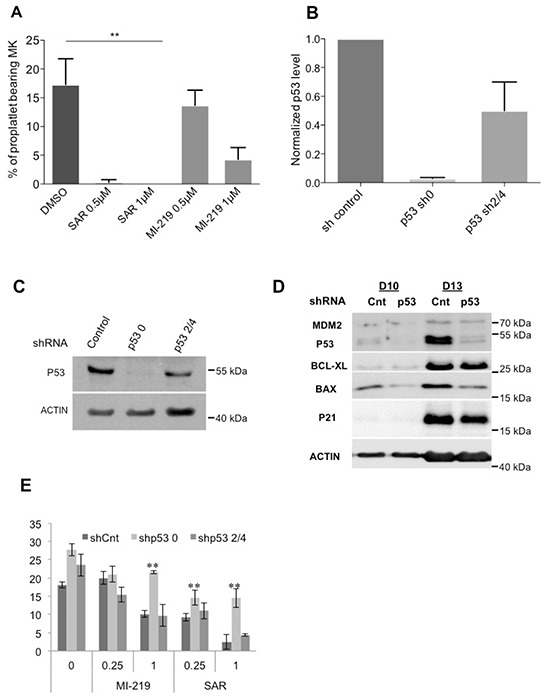
Effects of p53 stabilization on PPT formation **A.** Percentage of proplatelet-forming day 10-MKs after incubation with 0.5 or 1 μM of SAR405838 or MI-219. The percentage of proplatelet-forming MKs was estimated by counting MKs exhibiting one or more cytoplasmic processes with areas of constriction. A total of 200 cells per well were counted during 4 days. Error bars in histograms represent the SD of one representative experiment performed in triplicate wells. Similar results were obtained in 3 repeated experiments. * represents statistical significance (**P<0.01). **B.** Cells were transduced at day 4 of culture with a control lentivirus (shCnt) or a lentivirus encoding either shRNA p53 (shp53_0) or (shp53_2/4) and p53 gene expression levels were investigated by real-time PCR related to HPRT (n=5). **C.** p53 protein levels in GFP^++^CD41^+^ sorted cells were analyzed at day 9 of culture by western-blot. β-actin was used as loading control. **D.** p53, MDM2, BCL-XL, BAX and p21 protein levels were analyzed in GFP^+^/CD41^+^ sorted cells by Western blot (at days 10 and 13 of culture). β-actin was used as loading control. (n=3) * represents statistical significance (**P<0.01). **E.** Percentage of shCnt and shp53-transduced proplatelet-forming MKs exposed to 0.25 or 1 μM SAR405838 or MI-219. Day 8-MKs were sorted on the expression of GFP and CD41. Day 10-MKs were seeded with or without SAR405838 at 2×10^3^ cells/well in 96-well plate. A total of 200 cells per well were counted during 4 days (from day 11 to day 14). The histogram represents the percentage of proplatelet-forming MKs at day 12 of culture. (n=3) * represents statistical significance (**P<0.01).

Then, we explored the effects of p53 stabilization on PPT formation (the step before platelet release). On day 10-MKs, PPT formation was inhibited with 0.5 μM SAR405838 and completely abrogated at 1 μM (Figure 7A). To confirm that this was due to p53 stabilization, we knocked-down *p53* with 2 shRNA showing a decrease in *P53* RNA and protein levels (Figure 7B/7C) and reduced expression of p53 targets (Figure 7D). Both shRNA against *P53* significantly reverted the effect of SAR405838 on PPT formation (Figure 7E). This result clearly demonstrates that the drastic effect of SAR405838 on terminal MK differentiation is directly linked to p53 activation and not to an off-target effect.

## DISCUSSION

In this study, we showed that the MDM2/p53 axis is functional in hematopoietic progenitors and during erythroid and megakaryocytic differentiation. Moreover, p53 stabilization by MDM2 antagonists such as Nutlin, MI-219 [21] or a more potent one SAR40583, has a major deleterious effect on hematopoiesis. All the phenotypes observed in presence of SAR405838 were more pronounced than those with MI-219 or Nutlin that display lower binding affinities to MDM2 [9, 21].

Our results mainly show that SAR405838 induces a cytotoxic effect in all hematopoietic progenitors (erythroid, granulocytic, monocytic and megakaryocytic) including immature progenitors. SAR405838 induced not only a dose- but also a time-response cytotoxicity on progenitors suggesting that it is related to the proliferation and cell division. These results agree with those showing the deleterious effect of RG7112 on megakaryocytic progenitors [8]. Moreover, the effect on progenitors was irreversible since: i) the cytotoxic effect persisted even after the compound was washed and ii) high doses of TPO failed to revert the toxicity. Our results suggest that the use of TPO or TPO mimetics to rescue MDM2 antagonist-mediated thrombocytopenia would be difficult. However, only *in vivo* treatment may answer this question since TPO mimetics such as Romiplostim or Elthrombopag lead to substantial increased platelets in immune thrombocytopenia such as idiopathic thrombocytopenic purpura (ITP) or other cancer-related thrombocytopenia, even when a MK apoptosis is part of the mechanism of the thrombocytopenia [22, 23].

We have also shown that p53 reactivation induced by SAR405838, MI-219 or Nutlin affects early and late stages of megakaryopoiesis with a greater sensitivity on mature MKs than on MK progenitors. The sensitivity was clearly due to an increased apoptosis and a decrease in viable cells. Moreover, SAR405838 negatively impacts on the ploidization of MKs leading to hypoploid MKs. These effects of MDM2 antagonists on megakaryopoiesis are also quite similar to those of HDAC inhibitors, which also involve p53-dependent mechanism [24]. Finally, 0.5 μM SAR405838 drastically decreased while a 1 μM dose completely abolished the PPT formation. Moreover, the effect of MI-219 was less potent than SAR405838. These effects on MK differentiation completely matched with the results found for RG7112 on progenitors, maturation, ploidization and PPT formation during human megakaryocytic differentiation [8]. Our results are also in line with the fact that overexpressing p53 leads to a decreased polyploidization of the K562 human cell line after phorbol ester treatment [25]. Finally, the role of p53 in megakaryopoiesis has been nicely demonstrated in p53*^−/−^* mice. These mice present increased numbers of MKs with higher ploidy in stress conditions as well as platelet dysfunctions [26]. We may hypothesize that the mechanism by which a p53 increase interferes with polyploidization could be related to its control on either cell cycle checkpoints and/or apoptosis or the actomyosin cytoskeleton, or both. Comparative gene expression arrays in megakaryoblastic cell line CHFR depleted or not for p53 have highlighted “a megakaryocytic regulon of p53” with not only classical p53 target genes involved in apoptosis, DNA repair and cell cycle but also very interestingly non-p53 target genes implicated in the megakaryopoiesis such as actomyosin cytoskeleton components (MYH9, CTNNB1, FLA) [25].

We also observed that p53 reactivation induced by SAR405838 impacts other lineages such as the erythroid differentiation with a decrease in viable cells due to apoptosis. Mild anemia was observed in monkeys treated with RG7112 [8]. This finding correlates well with the role of MDM2/p53 in the regulation of erythropoiesis, which has been suggested by the defects in early and late erythropoiesis observed in mice with a conditional *Mdm2* knockout [27]. Noteworthy, the pathogenesis of the anemia in 5q- and Diamond–Blackfan syndromes could be related to p53 induction by a ribosomal stress [28–30].

The fact that SAR405838 displays a general toxicity on all hematopoietic progenitors and not specifically on MK progenitors seems in part contradictory to what was found in clinics since the patients harbor a strong thrombocytopenia associated or not with other cytopenia. Dissecting the blood parameters of the treated patients would be crucial to understand if there are real difference between *in vivo* and *in vitro* effects.

In clinics, the effect on thrombocytopenia seems reversible in a few weeks. Overall, the major hypothesis to explain the thrombocytopenia may be related to two different non-exclusive mechanisms: one on late stages of MK differentiation, particularly the PPT formation and another one on immature progenitors. However, depletion in immature progenitors would lead to a prolonged thrombocytopenia with a mild neutropenia and anemia. However, as this toxic effect requires several mitosis and early progenitors are mostly quiescent, this may suggest that MDM2 inhibitors mostly target mature progenitors. Therefore, one can speculate that the major effect of p53 reactivation *in vivo* is related to late stages of MK differentiation and platelet formation.

## MATERIALS AND METHODS

### Chemicals

SAR405838 and MI-219 were provided by Dr Laurent Debussche (Sanofi R&D Vitry-sur-Seine France). Nutlin3 a and b were a gift from Dr. Vassilev (Roche, Nutley, USA). Nutlin-3 was purchased from Calbiochem (Merck, Nottingham, UK).

### CD34^+^cell isolation

CD34^+^cells were obtained from leukapheresis samples after mobilization of donors after informed consent in agreement with our Institute Ethic Committee (Assistance Publique des Hôpitaux de Paris) and in accordance with the Declaration of Helsinki. CD34^+^cells were isolated by a positive selection using an immunomagnetic cell sorting system (AutoMacs; Miltenyi Biotec, Bergisch Gladbach, Germany).

### Hematopoietic progenitor cell assays

500 to 1000 normal CD34^+^cells were plated either in 1% methylcellulose assay (StemCells technologies, Vancouver, Canada) to quantify erythroid (BFU-E) and granulo-monocytic (CFU-GM) progenitors or in serum-free fibrin clot assay for quantification of MK progenitors (CFU-MK) in the absence or presence of various concentration of SAR405838 or MI-219. Cultures in methylcellulose were stimulated by addition of human recombinant growth factors: G-CSF (20 ng/mL), IL-3 (10 ng/mL) SCF (25 ng/mL, Biovitrum AB, Stockholm, Sweden), IL-6 (10 ng/mL), FLT3 (10 ng/mL) and human EPO (1 U/mL). Cultures in fibrin clot were stimulated with 10 ng/mL TPO (Kirin Brewery, Tokyo, Japan) and 25 ng/mL SCF. BFU-E and CFU-GM colonies were enumerated at day 14 of culture. CFU-MK colonies were enumerated at day 12 after labeling by an indirect immuno-alkaline phosphatase staining technique using an anti-CD41a monoclonal antibody (MoAb; Becton Dickinson, le Pont de Claix, France; clone HIP8), as previously described [31].

### Cell culture conditions

Normal CD34^+^ cells were cultured in serum-free medium supplemented with SCF (25 ng/mL), EPO (1 U/mL) and 15% of fetal calf serum for erythroblasts to induce erythroid differentiation. CD34^+^ cells were cultured in serum-free medium in the presence of TPO (10 ng/mL) and SCF (25 ng/mL) to induce MK differentiation.

### Lentivirus production

Lentiviruses encoding GFP and three different short hairpin RNA (shRNA) targeting p53 (0, 2 and 4) shRNA-0: *gactccagtggtaatctac* shRNA-2: *gagggatgtttgggagatg;* shRNA-4: *cggcgcacagaggaagaga* or a shRNA control were prepared and stored as previously described [32].

### Real time quantitative RT-PCR

PCRs were carried out in the ABI Prism GeneAmp 5700 (Perkin-Elmer) using the Power SYBR-Green PCR Master Mix (ABI) containing the specific primers. The expression levels of all genes were calculated relatively to *HPRT* or to *PPIA* housekeeping gene.

### Western blot analysis

Expression levels were performed by western-blot analysis using mouse anti-p53 and anti-β-actin (Sigma-Aldrich), anti-bax (Oncogene), anti-p21 (Beckton Dickenson), anti-Mdm2 (BD Pharmingen), anti-BCL-XL, and rat anti-HSC70 (Stressgen). Proteins were quantified by Image J software.

### Apoptosis assay

Apoptosis was measured by Annexin V-FITC detection kit (BD Pharmigen, San Diego, CA) and analyzed by flow cytometry on a LSRII (Becton Dickinson Mountain View, CA).

### Proplatelet formation assay

CD41^+^GFP^+^ MKs were sorted at day 8 of culture and plated in 96-well plates at a concentration of 2,000 cells/well in serum-free medium containing TPO, and quantification of PPT bearing MKs was performed as previously described [33]. The percentage of PPT bearing MKs was estimated by counting MKs exhibiting one or more cytoplasmic processes with areas of constriction. A total of 200 cells per well were counted during 4 days. Images were obtained using an inverted microscope (Carl Zeiss, Göttingen, Germany) at a magnification of 40× using the Axio Vision 4.6 software.

## SUPPLEMENTARY MATERIAL FIGURE


