# Supplementary material for: P53 activation inhibits all types of hematopoietic progenitors and all stages of megakaryopoiesis
Source: Oncotarget. 2016 Mar 3;7(22):31980–92. doi: 10.18632/oncotarget.7881 (PMC5077990; doi:10.18632/oncotarget.7881)
Supplement: Supplementary file 1 [file oncotarget-07-31980-s001.pdf]

P53 activation inhibits all types of hematopoietic progenitors and all stages of megakaryopoiesis

SUPPLEMENTARY FIGURE

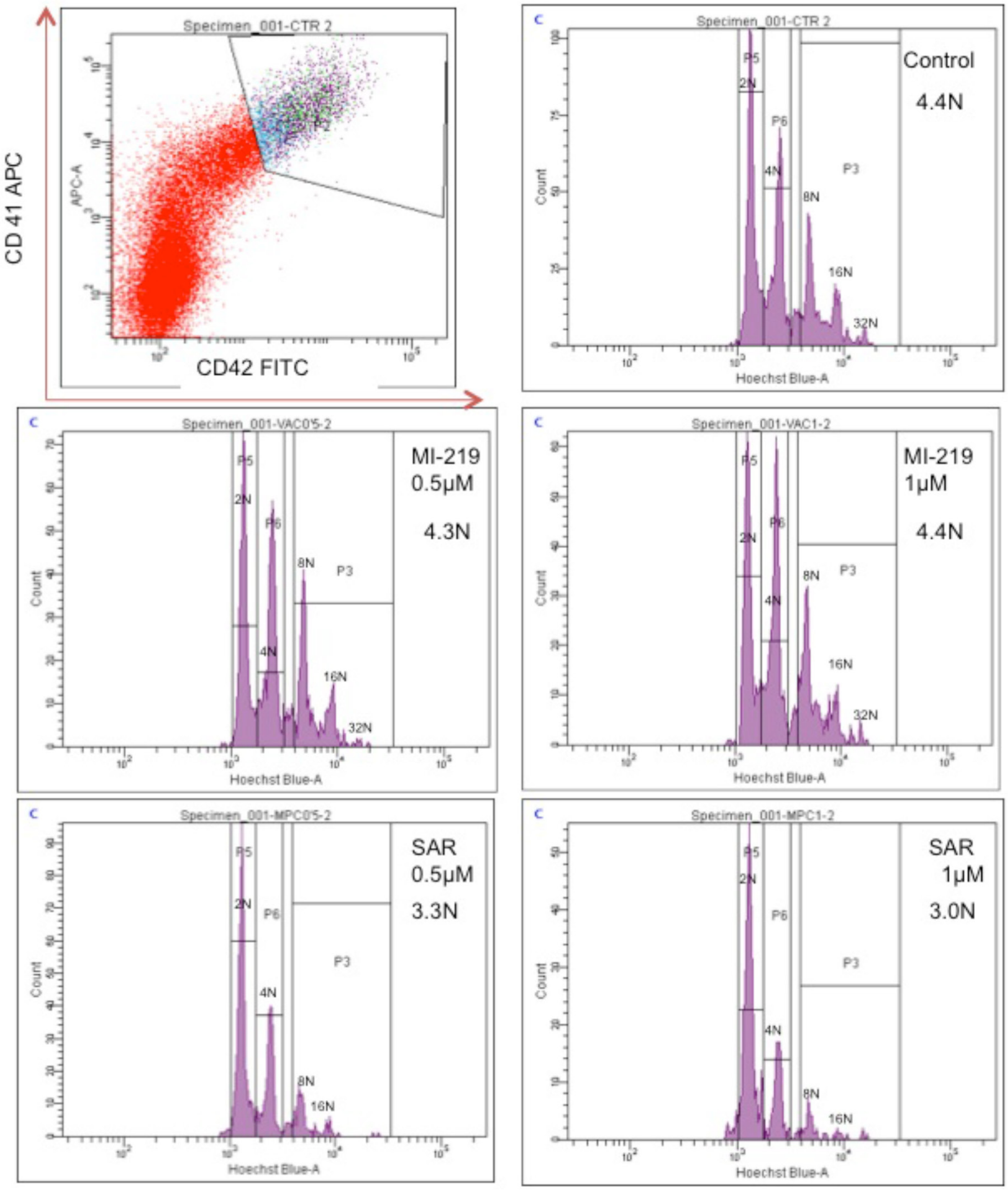

Supplementary Figure S1: Effect of SAR405838 on polyploidization. Day 6 megakaryoblasts were treated for 72 hours with various doses of MI-219 or SAR405838. At day 9, ploidy was analyzed by flow cytometry using a CD41/CD42/vital Hoechst staining. N represents the mean ploidy. As shown, SAR405838 induced a significant reduction in the mean ploidy (3.0N at the dose of 1µM versus 4.4N for control or for MI-219 treatment). The Figure illustrates a representative experiment in triplicate. Two other experiments in triplicate gave similar results.
